# Fabry Disease – Underestimated in the Differential Diagnosis of Multiple Sclerosis?

**DOI:** 10.1371/journal.pone.0071894

**Published:** 2013-08-28

**Authors:** Tobias Böttcher, Arndt Rolfs, Christian Tanislav, Andreas Bitsch, Wolfgang Köhler, Jens Gaedeke, Anne-Katrin Giese, Edwin H. Kolodny, Thomas Duning

**Affiliations:** 1 Albrecht Kossel Institute for Neuroregeneration, University of Rostock, Rostock, Germany; 2 Department of Neurology, Dietrich-Bonhoeffer-Klinikum, Neubrandenburg, Germany; 3 Department of Neurology, University Hospital, Giessen, Germany; 4 Department of Neurology, Ruppiner Kliniken GmbH, Neuruppin, Germany; 5 Department of Neurology, Fachkrankenhaus Hubertusburg, Wermsdorf, Germany; 6 Department of Nephrology, Charité Universitätsmedizin, Berlin, Germany; 7 Department of Neurology, New York University School of Medicine, New York, New York, United States of America; 8 Department of Neurology, University Hospital, Münster, Germany; Julius-Maximilians-Universität Würzburg, Germany

## Abstract

**Objective:**

Fabry disease is a rare X-linked inherited lysosomal storage disorder affecting multiple organ systems. It includes central nervous system involvement via micro- and macroangiopathic cerebral changes. Due to its clinical symptoms and frequent MRI lesions, Fabry disease is commonly misdiagnosed as multiple sclerosis. We present an overview of cases from Fabry centres in Germany initially misdiagnosed with multiple sclerosis and report the clinical, MR-tomographical, and laboratory findings.

**Methods:**

Eleven Fabry patients (one male, ten females) initially diagnosed with multiple sclerosis were identified from 187 patient records (5.9%) and analyzed for presenting symptoms, results of the initial diagnostic workup, and the clinical course of the disease.

**Results:**

Four patients were identified as having a “possible” history of MS, and 7 patients as “definite” cases of multiple sclerosis (revised McDonald criteria). On average, Fabry disease was diagnosed 8.2 years (±9.8 years) after the MS diagnosis, and 12.8 years after onset of first symptoms (±10.3 years). All patients revealed white matter lesions on MRI. The lesion pattern and results of cerebrospinal fluid examination were inconsistent and non-specific. White matter lesion volumes ranged from 8.9 mL to 34.8 mL (mean 17.8 mL±11.4 mL). There was no association between extra-neurological manifestations or enzyme activity and lesion load.

**Conclusion:**

There are several anamnestic and clinical hints indicating when Fabry disease should be considered a relevant differential diagnosis of multiple sclerosis, e.g. female patients with asymmetric, confluent white matter lesions on MRI, normal spinal MR imaging, ectatic vertebrobasilar arteries, proteinuria, or lack of intrathecally derived immunoglobulin synthesis.

## Introduction

Anderson-Fabry disease (Fabry disease) is an X-linked inherited lysosomal storage disorder which leads to a deficiency of a functionally active enzyme (α-galactosidase A – GLA) as a result of a defect in the GLA-gene [1]. This defect causes gradual deposition of neutral glycosphingolipids (globotriaosylceramide – Gb3) in various organ systems, resulting in a multi-system pathology including end-organ failure manifesting as hypertrophic cardiomyopathy and renal dysfunction [2]. Other clinical signs are angiokeratoma of the skin which was the topic of the original publications on this disease by William Anderson and Johannes Fabry in 1898 [3], [4]. Typical ophthalmological signs are cornea verticillata, retinal and conjunctival vascular changes, and cataract [5]. Gastrointestinal symptoms may be accompanied by diarrhoea and painful colic. Labyrinthine deafness or sudden loss of hearing occurs frequently. Although the clinical presentation of Fabry disease is very variable, neurological symptoms are the most common clinical feature and in most patients also the initial manifestations of Fabry disease [6], [7]. Episodes of severe neuropathic pain or paresthesia due to small fiber neuropathy, premature strokes, and cerebral white matter lesions are common manifestations and generally appear early in the course of the disease.

The involvement of the central nervous system (CNS) in Fabry disease is mainly due to cerebral vasculopathy, affecting both small and large cerebral vessels. Macroangiopathic alterations lead to an increased incidence of ischemic stroke, a major cause of the limited life span of Fabry patients. Apart from macroangiopathic changes, Fabry disease is frequently associated with early microangiopathic brain alterations with progressive white matter lesions (WML). At early stages, these lesions are not associated with clinical symptoms. Magnetic Resonance Imaging (MRI) studies have shown early and pronounced microangiopathic alterations of the brain associated with progressive WML in a large portion of Fabry patients of both genders (approximately 80%) [8]. In fact, there have been some case reports of children with Fabry disease and progressive WML [9], [10].

Other common findings are intermittent paresthesia in different locations and normal nerve conduction studies (due to the small fiber affection). Thus, in most cases, Magnetic Resonance (MR)-scans of the brain were conducted in Fabry patients to exclude CNS involvement that might cause the sensory deficits.

Available since 2001, enzyme replacement therapy (ERT) has been shown to clear globotriaosylceramide from the capillary endothelia, and has been demonstrated to reduce the severity of Fabry disease symptoms, offering the potential to significantly improve Fabry patients' quality of life [11]. Existing symptoms may regress, especially if ERT is started early − ahead of the development of irreversible organ damage [12]. ERT also reduces the risk of developing further organ damage. Conversely, manifest organ damage is usually irreversible if treatment is started late, and thus early diagnosis of Fabry disease is essential. However, due to the variable clinical symptoms, the diagnosis of Fabry disease is challenging. To date, the mean delay between first symptoms and diagnosis is 10 to 14 years for affected males, and 10 to 16 years for women [2]. Common misdiagnoses are rheumatic fever or other rheumatic diseases (especially in children), somatoform pain disorder and collagen vascular disease [13], [14], [15].

Taking into account the clinical presentation and the common MRI lesions, it is understandable that conflicting considerations may occur in the scope of Fabry disease and multiple sclerosis (MS) [16], [17], [18], [19], although cases of misdiagnosis of MS in Fabry patients have already been described in pre-MRI times [20]. Due to the combination of intermittent (“relapsing”) sensory deficits and disseminated WMLs found in patients with Fabry disease, they might in fact fulfill the revised McDonald criteria [21], [22], [23] for “possible” or even “definite” MS. Additionally, a false diagnosis can be accelerated by the misinterpretation of cerebrospinal fluid (CSF) abnormalities observed in some Fabry patients [24]. The following study provides an overview of Fabry cases initially misdiagnosed as MS from Fabry centres in Germany. We report the typical clinical, MR-tomographical, and laboratory findings and suggest red flags that should trigger a specific Fabry disease diagnostic workup.

## Methods

### Ethics Statement

The present case series was performed as a subproject to the ongoing study “Prevalence of Fabry Disease in a Defined Population at Risk – Patients formerly Diagnosed with Multiple Sclerosis” (ClinicalTrials.gov Identifier NCT 01271699). Both the principal study and the subproject were approved by the Ethics Committee of the University of Rostock. Written informed consent was obtained from all patients included in this case series.

Fabry patients formerly diagnosed with MS were indentified from the patients' charts and records at the centres involved and thoroughly analysed for their presenting symptoms, the results of the initial diagnostic workup and the clinical course of their disease.

Inclusion criteria were:

Genetically proven Fabry disease
Former diagnosis of “possible” or “definite” MS in accordance to the revised McDonald criteria [21], [22], [23]
Completion of MRI of the brain and spinal cord (minimum: standard T1- and T2-weighted images, gadolinium enhanced T1-weighted images as well as FLAIR-sequences)
Completion of lumbar puncture and CSF analysis
No evidence of other neurological diseases.

### MRI data analysis

Volumetry of WML was performed by two trained, independent and experienced operators, who were blinded to clinical data. Lesion load on axial FLAIR sequences was determined in a semiautomated way by outlining the peripheral borders of white matter lesions. Lesions were marked and borders were set by local thresholding using a customized software based on Analyse software (Brain Imaging Resource, Mayo Clinic, Rochester, MI, USA). Whenever necessary, borders could be adjusted by the operators by changing the thresholds for upper and lower intensity values. Following delineation of all lesions, the program calculates the total surface of the outlined area. By multiplication with the interslice distance, total volume of WML is established. Intra- and interobserver reliability was good with a weighted Kappa of 0.98 and 0.93 respectively.

### Statistical analysis

Spearman correlations were used to assess associations between white matter lesion volumes and clinical measures (Glomerular filtration rate – GFR, proteinuria, end-diastolic left ventricular wall thickness, NYHA score and neuropathic pain score) and laboratory values (GLA activity, Gb3 and lyso-Gb3 level). Data analysis was carried out using SPSS 15.0.0. The significance level was set at P<0.05.

### Results

In summary, documentation and recordings of clinical data of 187 patients with genetically proven Fabry disease were screened. Eleven of these patients (5.9%) could be identified at the participating centres as fulfilling the entry criteria (one male, ten females). Demographical data as well as presenting symptoms are depicted in Table 1. Time from onset of first symptoms and diagnosis of MS was 4.4±3.4 years. Four patients were identified as “possible” MS in accordance to the revised McDonald criteria, 7 patients as “definite” MS. Seven out of 11 patients received MS-specific treatment (two patients with i.v. steroids only, and 5 patients with interferone i.m. or s.c., or glatirameracetate). Fabry disease was diagnosed on average 8.2 years (±9.8 years) after diagnosis of MS, and 12.8 years after onset of first symptoms (±10.3 years).

**Table 1:** Demographic data and clinical presentation.

Pat. No	Sex	Age [y]	First sym ptoms at age [y]	Initial symptoms	MS Diagnosis at age [y]	Fabry Diagnosis at age [y]	ERT initiated at age [y]	Initial respon se to steroids	EDSS (initial/ now)	McDonald cri teria (MS/possi ble/not MS)	MS-specific treatment
01	f	39	35	Postpartum transient vertigo and double vision	37	38	38	No steroid treatment	1.0/n.d.	Possible MS	non
02	m	30	21	Subacute vertigo, double vision, slight paresis of the left leg	25	26	26	Yes	3.0/8.0	MS	Glatirameracetate s.c.
03	f	47	37	Headache, vertigo, cognitive deficits, intermittent double vision	41	45	45	No steroid treatment	1.5/n.d.	MS	Interferon sc.
04	f	56	39	Spastic paraparesis, gait disturbances	39	53	53	Yes	3.0/7.5	MS	Steroids i.v., Interferon s.c.
05	f	60	48	Paresis of the left arm, paraesthesia right leg	50	55	55	No	2.5/3.0	Possible MS	Steroids i.v.
06	f	43	24	Spastic hemiparesis, ataxia, distal paraesthesia arms and legs	29	34	39	Yes	3.0/3.5	MS	Steroids i.v., Interferon s.c.
07	f	60	48	Spasmus hemifacialis, paresthesia of the right arm	53	57	57	No	1.0/1.5	MS	non
08	f	28	14	Visual disturbances	27	28	Not yet indicated	No steroid treatment	0.0/n.d.	Possible MS	non
09	F	46	44	Alternating paraesthesia left arm and left leg	44	46	not indicated	n.a.	2.0/2.0	Possible MS	non
10	F	64	24	Alternating sensori motor paresis right arm, ataxia	29	56	56	Yes	3.0/5.5	MS	Steroids i.v. and oral
11	F	68	28	Chronic fatigue, alternating paraesthe sia and mild paresis of right arm	34	60	60	No	1.5/1.5	Possible MS	Steroids i.v. and oral

MS  =  multiple sclerosis; ERT  =  enzyme replacement therapy; EDSS  =  expanded disability status scale; i.v.  =  intravenous; s.c.  =  subcutaneous; n.d.  =  not done; n.a.  =  not available.

The average clinical stage of patients was mild to moderate; three patients (patients 2, 6 and 10) were severely affected. Acroparaesthesia was a common complaint, and neurological features were also the earliest to develop. However, classical episodes with neuropathic pain were reported in only 7 cases (64%). Manifest cerebrovascular events (strokes or intracerebral hemorrhage) were not reported in any patient. An elevated left ventricular wall thickness as typical cardiac presentation of Fabry disease could be detected in 7 cases. Classical renal involvement associated with presence of proteinuria and/or GFR reduction was present in 8 patients, but end-stage renal failure with the need of hemodialysis was present in none of these cases.

**Figure 1:**
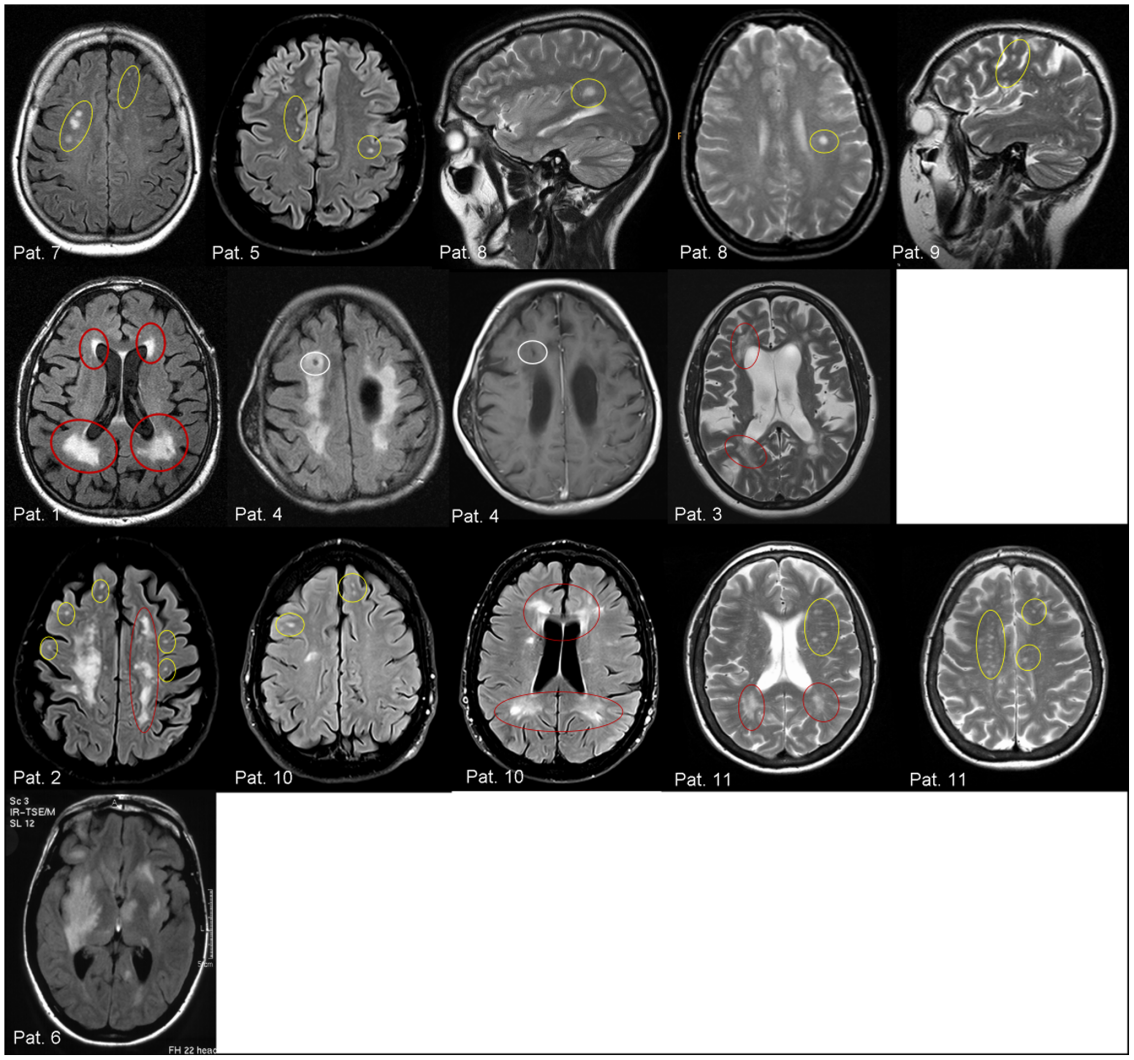
MR images (T2-weighted and FLAIR sequences) of 11 Fabry patients misdiagnosed with multiple sclerosis. *Upper row:* Patients 7, 5, 8 and 9 showed more punctuate and subcortical white matter lesions (yellow circles). *Second row:* White matter lesions in patients 1, 4, and 3 had a rather confluent, periventricular pattern (red circles). This pattern was associated with “black holes” (white circles) in T1-weighted images as a surrogate of severe demyelination and axonal injury. *Third row:* Patients 2, 10, and 11 revealed both disseminated and confluent, periventricular patterns. *Fourth row:* Only patient 6 showed an uncommon image with a severe, asymmetrical involvement of cortical, subcortical, and basal ganglia tissues.

MRI scans of all patients revealed cerebral white matter lesions. Localization of the WML was primarily subcortical, with punctuate lesions in 4 cases. 3 patients showed mainly periventricular confluent WML. Three other patients had both periventricular and subcortical WML localizations. For one patient (no. 6), an uncommon MR image documenting a severe asymmetrical involvement of the cortical, subcortical, and basal ganglia tissues was derived (Figure 1). Gadolinium enhancement was not observed in any of the cases. MRI scans of the spinal cord were unrevealing in all cases.

WML load ranged from 8.9 mL to 34.8 mL (mean 17.8 mL±11.4 mL) and was significantly higher in patients demonstrating periventricular lesions. There was no association between cardiac or renal involvement (p>0.05) on one hand and white matter lesion volumes on the other, despite a trend towards decreased renal function (GFR values) in patients with higher lesion loads (p = 0.071). Also, neuropathic pain scores or laboratory values (GLA-activity and lyso-Gb3 level) and lesion volumes were not significantly correlated. The basilar artery diameter has recently been reported as a useful screening tool for Fabry disease in young stroke patients, with a sensitivity of 84% [25]. In contrast, we found no ectatic vertebrobasilar arteries or enlarged basilar artery diameters in our cohort.

Findings in CSF analysis revealed inconsistent and non-specific results. Four patients showed mild pleocytosis in the CSF analysis (>5 up to 35 cells/µL). Results for CSF cytology were available for 6 patients and mostly revealed a lymphocytic reaction. Only in one patient, no. 10, granulocytic pleocytosis was found. Slightly increased protein levels, indicating a dysfunction of the blood cerebrospinal fluid barrier (BCSFB), were found in 3 patients (maximum 870 mg/L). Protein levels increased when CSF analysis was conducted consecutively (e.g. in patient 11), most likely due to the progression of white matter damage. Only one patient (patient 3) revealed intrathecal immunoglobulin synthesis and positive oligoclonal bands. Detailed results of the CSF analysis are presented in Table 2.

**Table 2:** CSF results of first diagnostic spinal tap.

Patient No	Cell count (cells/µL; Nor mal value: <5)	CSF cytology	Total protein (mg/L; Normal value: <450)	Intrathecal Ig- synthesis (Reiber formula)	Oligoclonal bands	Comments
01	17	n.a.	330	No	Borderline (2 bands)	
02	25	n.a.	437	No	Absent	Similar results in multiple CSF analysis
03	10	94% lymphocytes, 4% monocytes, 0.2% plasma cells	870	IgG 23%, IgA 7%	Positive	
04	4	95% lymphocytes, 2% monocytes, 3% granulocytes	335	No	Absent	Negative for antibodies against Borrelia burgdorferi, herpes virus, and Treponema pallidum
05	1	n/a	430	No	Absent	Negative for antibodies against Borrelia burgdorferi, normal levels of angiotensin-converting enzyme
06	0	n/a	396	No	Absent	Same results in multiple CSF analyses
07	5	78% lymphocytes, 22% monocytes	n.d.; albumin 192 mg/l; no BCSFB failure	No	Absent	Similar cell count in two following CSF analyses
08	1	97% lymphocytes, 3% monocytes	230	No	Absent	
09	4	n.a	440	No	Absent	Negative for antibodies against Borrelia burgdorferi, normal levels of angiotensin-converting enzyme
10	35	30% lymphocytes, 62% granulocytes	740	No	Absent	Similar results in three successive CSF analyses
11	3	44% lymphocytes, 54% granulocytes	520	No	Absent	Similar results in multiple CSF analyses, but protein levels increased up to 688 mg/L (last CSF analysis in 2009)

BCSFB  =  blood cerebrospinal fluid barrier; CSF  =  cerebrospinal fluid; n.d.  =  not done; n.a.  =  not available.

Genetic study of the GLA-gene, examination of GLA enzyme activity and analysis of lyso-Gb3 plasma levels yielded results not deviating from routine diagnostic findings in Fabry disease, with a broad spectrum of different mutations and highly diverse biochemical findings. These aspects and the results of the diagnostic workup (including renal and cardiac affection) are depicted in Table 3.

**Table 3:** Fabry-specific symptoms and results of genetic analysis.

Pat. No	Neuropathic pain	Renal manifestation		Cardiac involvement		GLA-gene mutation	GLA leukocyte enzyme activity (nmol mU/ mg; norm ≥5.0)	Lyso Gb3 plasma level (ng/mL; norm ≤0.40)
		GFR (ml/min)	Proteinuria	LVWT (mm)	NYHA			
01	0	>60	+	0	0	c.1240-1241delTT	6.0	5.77
02	+	>60	+++	0	0	c.1246C>T [Q416X]	13.0	25.9
03	0	>60	0	++	II	c.902G>A [R301Q]	n.a.	2.45
04	0	>60	0	+	II	c.658C>T [R220X]	55.0	3.35
05	+	48	+	+	I	IVS2 -1C>T	n.a.	n.a.
06	++	24	+++	++	III	c.706T>C [W236R]	7.4	n.a.
07	++	37.7	++	+	I	c.424T>C [C142R]	5.0	0.79
08	0	>60	+	0	0	c.376A>G [S126G]	38.0	0.42
09	+	>60	0	0	0	c.427G>A [A143T]	n.a.	<LOD
10	+	34	++	++	III	c.281G>C [C94S]	76.0	1.73
11	+	44	+	+	I	c.C679>T [R227X]	2.54	n.a.

Neuropathic pain: 0  =  no pain; +  =  mild to moderate pain; ++ typical severe neuropathic pain with pain attacks.

Proteinuria: 0 = <30 mg/dL; +  = 30–300 mg/dL; ++  = 301–1000 mg/dL; +++  =  >1000 mg/dL.

LVWT: end-diastolic left ventricular wall thickness; 0 = 6–9 mm (normal); +  = 11–13 mm (mild); ++  = 14–17 mm (moderate); +++  = >17 mm (severe).

NYHA: New York Heart Association Functional Classification; GFR: Glomerular filtration rate; GLA  =  alpha-Galactosidase; Gb3  =  Globotriaosylceramide; n.a.  =  not available; LOD  =  limit of detection.

## Discussion

The diagnosis of MS, one of the most important and most frequent neurological disorders in Western countries which causes permanent functional deficits in young patients, requires a detailed workup to exclude any relevant concurring inflammatory and non-inflammatory diseases mimicking clinical and/or imaging features associated with MS. Among the differential diagnoses to be considered are some hereditary disorders, especially Fabry disease. Like MS, Fabry disease affects young people and can develop monosymptomatic courses which only involve the CNS without any medical history of classical Fabry symptoms such as angiokeratoma, cornea verticillata or heart and kidney diseases. We present several cases initially diagnosed with MS and later proved positive for Fabry disease. In all likelihood, it was the coexistence of neurological deficits, cerebral MRI abnormalities, and, in some cases, conspicuous CSF results which gave rise to misinterpretations.

A common feature in most of the initially misdiagnosed Fabry patients is the overrepresentation of cerebellar and brain stem symptoms (e.g. ataxia, gait disturbances, double vision, vertigo) attributable to vertebrobasilar vascular territory (for details see Table 1). Although we did not find any evidence of manifest cerebral infarction or WML within the brainstem or cerebellum in our patients, these symptoms most likely must be considered as transient ischemic attacks, which is in good accordance with the reported clinical registry data of Fabry patients [6], [15]. In most cases, the symptoms appeared intermittently and, in combination with the common presentation of sensory deficits (due to small fiber neuropathy) and the MRI findings, can easily mimic MS by fulfilling the criteria of spatial and temporal dissemination. Vertigo, diplopia, dysarthria and ataxia, however, may also occur in early MS, although typical features such as an initial presentation with optic neuritis or internuclear ophthalmoplegia were not present in our cases. It is important to note that most of the false MS diagnoses were made applying the original McDonald criteria [21] or their first revision [22]. The potential risk of misdiagnosis might even be higher for the criteria of the latest revision [23], since its definition of dissemination in space and time is less stringent. Additionally, it is possible to arrive at a “definite” or at least “possible” diagnosis of multiple sclerosis even without any examination of the CSF at all. Since only one misdiagnosed Fabry patient revealed oligoclonal bands in CSF analysis, these findings might be uncommon in Fabry disease, and CSF analysis could help to differentiate between MS and Fabry disease.

Using the revised diagnostic criteria, diagnosis of MS in Fabry patients was mainly supported by MRI findings. We found two characteristic patterns of WML in our patients: four of the patients had more punctuated and subcortical white matter lesions and thus presented a typical disseminated pattern. In contrast to typical MS lesions, however, these lesions were not particularly located within the *corpus callosum*, and none of the Fabry disease patients showed MS-typical demyelinating plaques across the *corpus callosum* (“Dawson fingers”). The second group of Fabry patients revealed a rather confluent periventricular pattern (red circles in Figure 1) of WML. These lesions were often associated with “black holes” (white circles) in T1-weighted images as a surrogate of severe demyelination and axonal injury and were also common in advanced MS. Although it is tempting to speculate that these lesions occurred in later and more severe stages of Fabry disease, possibly following the punctuated WML, we failed to establish any association with systemic organ involvement or with the duration of the disease. Thus, the underlying pathomechnisms of renal or heart damage might differ from the microangiopathic cerebral lesions in Fabry disease, and makes it even more difficult to differentiate between Fabry disease and MS.

Several studies documented a higher frequency of ischemic lesions in the vertebrobasilar territory in Fabry patients, correlated with more pronounced changes in the vertebrobasilar vessels [26], [27]. In most cases, these occipito-parietal lesions were indicated as embolic events [28]. However, although cerebellar and brain stem symptoms commonly appeared, the WML in our patients were not clustered in the occipitoparietal areas. These findings underline the fact that there are two types of cerebral damage in Fabry patients: embolic strokes and microangiopathic lesions, and that these lesion types are not directly associated.

Spinal MR imaging was consistently normal in our cohort. Only one Fabry patient previously described [18] had hyperintense T2-lesions in the cervical or thoracic spine suspicious of MS lesions. Thus, spinal lesion seems to be an uncommon manifestation in Fabry patients, and spinal MR could help to distinguish between MS and Fabry disease. However, one possible limitation should be noted: Fabry patients are not regularly subjected to spinal MR. Thus, spinal lesions might be underestimated in Fabry disease.

Reports of CSF abnormalities in Fabry disease described an “aseptic meningitis” or “chronic meningitis” with mild to moderate pleocytosis (up to 76 cells/µL) and slightly elevated total protein levels (up to 800 mg/L), suggestive of a disturbed BCSFB function [24], [29]. Interestingly, high percentages of neutrophil granulocytes were observed which sometimes exceeded 30% [30], [31], [32]. Similar results were obtained in a minority of our patients only. Pleocytosis was present in 4/11 cases, while dysfunction of BCSFB was detected in 3/11 cases. CSF cytology was reported in 6/11 of the patients and revealed substantial granulocytic involvement in two cases. Autoinflammatory processes, as discussed in the scope of Fabry disease [24], usually go along with lympho-monocytic responses. Polymorphic nuclear granulocytes are attracted either by infectious agents or by foreign stimuli like blood (e.g. subarachnoid haemorrhage) or implanted devices. Results from post-mortem studies in two Fabry patients describe a highly abundant accumulation of ceramide trihexosides in the choroid plexus and leptomeninges [33]. This offers the hypothesis of an aseptic inflammatory process triggered by the stored lipids acting as a foreign body stimulus. Nevertheless, mild CSF pleocytosis does not necessarily suggest inflammatory processes but can also be found after brain infarctions.

Regarding the initial diagnosis of MS, another CSF feature is even more surprising: the overwhelming lack of intrathecally derived immunoglobulin (Ig) synthesis, proved by an increased IgG fraction in the Reiber formula, or by detection of oligoclonal bands. Detection of oligoclonal bands was reported in only one previous case [18] and also in only 1/11 of our patients. One has to take into account that oligoclonal bands, although very sensitive in MS, are highly unspecific and can occur even after unspecific infections. In fact, the above-mentioned pathophysiological considerations might explain intrathecal immunoglobulin synthesis in some cases, but it seems to be uncommon in Fabry disease and could help to distinguish between both diseaes.

Despite its X-chromosomal inheritance, Fabry disease also causes clinical symptoms in a high percentage of affected females, due to the phenomenon of lyonization which describes the random tissue-dependent inactivation of one X allele [34]. Whereas male patients usually develop the full expression of the condition, the course in women is highly variable [35]. In a recent analysis of Fabry registry data, Sims et al. reported that ischemic cerebral lesions frequently occur in the absence of clinical events before the diagnosis is made [6]. Cerebral involvement can be the first and only symptom of Fabry disease. Interestingly, especially female patients had not experienced renal or cardiac events before a cerebral affection occurred (77% of female Fabry patients). When looking at Fabry patients under 30 years of age, particularly females were more likely to have experienced cerebral affection as their only clinical manifestation (67% of females). These results indicate that in particular women with Fabry disease might develop a rather monosymptomatic course of the disease, leading to primary cerebral affection. This might explain the gender distribution in our patient cohort and suggests that Fabry disease should particularly be considered in female patients with “atypical” MS. Since the female-to-male ratio of early diseased patients with MS is about 3∶1, the gender ratio is another similarity between MS and Fabry disease and may also complicate the differentiation between these two diseases.

There is no doubt that distinguishing multiple sclerosis from Fabry disease can be challenging under certain circumstances, especially in the absence or only suble presence of non-neurological manifestations of Fabry disease (e.g. renal or cardiac involvement, angiokeratomas, etc.). Based on our own cases and descriptions from the literature, however, we come to the conclusion that there are some critical parameters in the diagnostic workup for multiple sclerosis. Fabry disease should be seriously considered for:

female patients with

asymmetric, confluent white matter lesions on MRI without or with very mild corpus callosum T2-hyperintensities and/or
lack of gadolinium enhancement and/or normal spinal MR imaging and/or
ectatic vertebrobasilar arteries and/or
lacking evidence of intrathecally derived immunoglobulin synthesis (diagnostic standard: oligoclonal bands)
significant proteinuria (urine dipstick test) or unexplained left ventricular hypertrophy
relatives who died at a young age from unspecified renal, cardiac or cerebrovascular disease (X-linked).

Despite the limited number of patients examined, the arguments outlined in this study highlight that in view of ERT being available as an effective and well-tolerated causal therapeutic option for Fabry disease, this diagnosis should not be missed. Besides possible complications of ineffective and unnecessary MS treatment, patients might suffer organ damage, in some cases irreversible, as a result of delaying the start of enzyme replacement therapy.
